# *Wickerhamomyces anomalus* in Mosquitoes: A Promising Yeast-Based Tool for the “Symbiotic Control” of Mosquito-Borne Diseases

**DOI:** 10.3389/fmicb.2020.621605

**Published:** 2021-01-21

**Authors:** Alessia Cappelli, Guido Favia, Irene Ricci

**Affiliations:** School of Biosciences and Veterinary Medicine, University of Camerino, CIRM Italian Malaria Network, Camerino, Italy

**Keywords:** Wickerhamomyces anomalus, yeast, insect, mosquito, malaria, symbiosis, mycobiota, symbiotic control

## Abstract

The ascomycete yeast *Wickerhamomyces anomalus* is a mutualistic symbiont of different insects, including diptera vectors of diseases. Although fungal symbioses have been so far poorly characterized, the topic is gaining attention as yeast-insect interactions can provide pivotal information on insect biology, such as their environmental adaptation or vectorial capability. We review the symbiosis between *W. anomalus* and mosquitoes, which implies nutritional and protective functions. Furthermore, we focus on antiplasmodial effects of *W. anomalus* in malaria vectors and discuss the yeast potential for the “symbiotic control” (SC) of mosquito-borne diseases (MBDs).

## Introduction

Mosquitoes comprise different species that are vectors of pathogens to humans and other animals. The most important mosquito-borne diseases (MBDs) are transmitted by three mosquito genera: *Aedes*, *Anopheles*, and *Culex*. *Aedes* transmit several arboviruses including Dengue, Yellow Fever, Chikungunya, Zika, and West Nile; *Anopheles* spread malaria parasites (*Plasmodium* spp.); *Culex* is a major vector of West Nile virus and filarial nematodes. In the last decades, climate changes, anthropization of new habitats, and international trade have favored the global expansion of mosquito vectors and emergence of MBDs causes major public health concerns in new geographic areas (Brugueras et al., 2020). Since vaccines against most mosquito-borne pathogens are not available, their prevention relies mainly on insecticides. However, insecticides become less effective as vectors develop resistance and the overuse of chemical insecticides increases the costs besides having deleterious effects on non-target species and the environment. Thus, innovative eco-friendly alternatives are requested. New control methods are focusing on the rising knowledge of the mosquito microbial community (microbiota) and its effect on the mosquito-pathogen relationship. Understanding the biology of symbionts in insect vectors is essential for the development of biological control strategies like the “symbiotic control” (SC; Bourtzis et al., 2014). The SC is a promising non-chemical method for the control of vectors and diseases they transmit. This approach exploits symbiotic microorganisms, bacteria or fungi, with the aim of reducing the vector capability (Niang et al., 2018). One of the strategies for the SC of vector-borne pathogens is based on the exploitation of antagonism between symbionts and pathogens, as in the case of the bacteria *Wolbachia* that is applied for the control of mosquito-borne viruses (Frentiu, 2017).

The analysis of the mosquito-associated microbiota (bacteria, fungi, protists, viruses, and nematodes) is gaining attention since microbes are recognized to influence many traits of the mosquito biology, such as development, physiology, immunity, and vector competence (Guégan et al., 2018). While an increasing number of studies have focused on bacteria, the fungal community (mycobiota) has been largely neglected, but recent studies show the presence of an important fungal diversity in mosquitoes (Malassigné et al., 2020). Mosquito-mycobiota is mainly composed of Ascomycota, which comprise mostly species of Pezizomycotina and Saccharomycotina (subphyla); Pezizomycotina include species of filamentous fungi, whereas Saccharomycotina are basically represented by yeasts, such as *Candida*, *Meyerozyma*, *Pichia*, and *Wickerhamomyces*, that adapt to survive in the insect gut and different mutualistic yeast-insect symbioses based on trophic interdependence have been described (Malassigné et al., 2020). Yeasts generate signals of sugar resources through metabolic pathways that produce compounds, such as fermentative volatiles, that attract insects. On the other hand, insects disperse yeasts over a variety of sugar sources and provide them with food and a habitat (the gut) to mate or generate sexual forms, increasing yeast biodiversity (Madden et al., 2018). Yeasts are important not only for attraction to food, they influence oviposition sites and larval development, but also supply diet integration of adults providing organic nitrogen, essential vitamins, and lipids (Stefanini, 2018). In summary, insects are an essential component of the ecology of ascomycetous yeasts, and the latter influence many traits of the insects’ biology.

*Wickerhamomyces anomalus* (class: saccharomycetes) is a budding yeast identified in the gut of diverse insects, in which it is suggested as a mutualist symbiont with nutritional and protective functions (Toki et al., 2013; Steyn et al., 2016). The presence of *W. anomalus* is reported in different orders of insects, such as mosquitoes (Diptera: Culicidae), sand flies (Diptera: Psychodidae), honey bees (Hymenoptera), plant hoppers (Hemiptera), and beetles (Coleoptera). Several studies focused on the yeast association with mosquitoes, in which *W. anomalus* is identified in *Anopheles*, *Aedes*, and *Culex* species (Ricci et al., 2011a; Muturi et al., 2016; Steyn et al., 2016). Interestingly, the strain of *W. anomalus* identified in the malaria vector *Anopheles stephensi* produces a killer toxin (KT) with wide antimicrobial activities that may protect the host from entomopathogenic microbes (Ricci et al., 2011b). KTs are glycoproteins with exo-β-1,3-glucanase enzymatic activity that hydrolyze glucans on the surface of different microbial targets, such as viruses, bacteria, fungi, and protozoa (Walker, 2011). Additional studies demonstrated a strong KT-based effect against the protozoa *Plasmodium berghei* that prevent the parasite development in the midgut of *An. stephensi* (Cappelli et al., 2019). Here, we review the *W. anomalus* symbiotic association with mosquitoes and focus on the antiplasmodial effect, discussing the potential application of a new yeast-based approach for the SC of malaria and possibly other MBDs.

## General Features of *Wickerhamomyces anomalus*

*Wickerhamomyces anomalus*, formerly *Pichia anomala* and *Hansenula anomala*, was renamed following phylogenetic studies (Daniel et al., 2012). The biotechnological potential of this yeast and its use in different industrial applications derive from capability of *W. anomalus* to secrete antimicrobials factors acting on a broad spectrum of pathogens (Walker, 2011). In the last decade, the yeast has drawn special attention and the genome sequences of two environmental strains have been published (Schneider et al., 2012; Cunha et al., 2020). The European Food Safety Authority (EFSA) classifies *W. anomalus* at biosafety level one and different “killer strains” (yeast isolates that produce KTs) are used as biocontrol agents with inhibitory effects against mold and bacteria in the agro-food sector (Sundh and Melin, 2011). Specific properties make *W. anomalus* a suitable product for different biotechnological applications: (i) adaptation to a wide range of growth conditions in terms of temperature (3–37°C), pH value (2–12), and osmolarity; (ii) robustness and competitiveness in different habitats; (iii) ability to produce biomass on a large scale; (iv) stress-tolerance, survival, and maintenance of biocontrol activity in different formulations and after desiccation (Passoth et al., 2011).

*Wickerhamomyces anomalus* has been isolated from different environmental matrices: flowers and leaves, food and feed systems, and waters and insects (Walker, 2011). Moreover, opportunistic strains have been detected in immunocompromised patients and, even though few outbreaks have been reported, they should be taken into consideration (Dutra et al., 2020). Thus, distinguishing pathogenic from non-pathogenic strains is crucial for future applications of *W. anomalus*. Since it was identified in hematophagous insects (mosquitoes and sand flies), a study investigated whether *W. anomalus* infects humans: screening immunocompromised and malaria patients, and healthy volunteers (exposed to mosquito bites) revealed that *W. anomalus* is not relevant to human, consistently with the rare reports of fungemia (Epis et al., 2015). Safety tests showed that *W. anomalus* does not harm mammalian cells, suggesting that they are not sensible to the yeast killer activity (Cappelli et al., 2019).

## *Wickerhamomyces anomalus*-Mosquito Symbioses and Their Impact on the Insect Biology

On the background that *W. anomalus* has been isolated from different environmental matrices such as flowers and water, mosquitoes larvae can acquire the yeast from the aquatic breeding sites, whereas adults by feeding on nectars. First identifications of *W. anomalus* in mosquitoes were in *Anopheles stephensi* (lab-reared colony), and *Anopheles gambiae*, *Aedes aegypti*, and *Aedes albopictus* (lab-reared colonies and wild samples; Ricci et al., 2011a,b). An in-depth screening of the mycobiota in *Culex* spp. revealed the presence of *W. anomalus* in *Culex pipens* (wild samples; Steyn et al., 2016). More recent investigations by metagenomic analyses of invasive species identified the yeast in *Aedes japonicus* and *Aedes triseriatus* (wild samples; Muturi et al., 2016). However, mosquitoes are not the only host of *W. anomalus* since it is found and suggested as mutualist symbiont in *Phlebotomus perniciosus* (sand fly), *Apis mellifera* (honeybee), *Laodelphax striatellatus* (planthopper), and *Doubledaya bucculenta* (beetle; Table 1).

**Table 1:** *Wickerhamomyces anomalus* in mosquitoes and other insects.

Insect host (order)	Species	Symbiotic functions described (bold) or proposed (?)	Developmental stages (organs)	References
Mosquito (Diptera: Culicidae)	*Anopheles stephensi* (laboratory reared colonies)	**Protective role: killer strain with antifungal and antiplasmodial effects (b-1,3-glucanase activity)**; Nutritional role in larvae and adults (?)	Larvae, pupae, adult male, and female (midgut, gonads)	Ricci et al., 2011b; Cappelli et al., 2014, 2019; Valzano et al., 2016; Cecarini et al., 2019
	*Anopheles gambiae* (wild samples and laboratory reared colonies)	Nutritional/protective role (?)	Adult male and female	Ricci et al., 2011a
	*Aedes albopictus* (wild samples and laboratory reared colonies)	Nutritional/protective role (?)	Adult male and female	Ricci et al., 2011a
	*Aedes aegypti* (wild samples and laboratory reared colonies)	Nutritional/protective role (?)	Adult male and female	Ricci et al., 2011a
	*Aedes japonicus* (wild samples)	Nutritional/protective role (?)	Adult female (midgut)	Muturi et al., 2016
	*Aedes triseriatus (wild samples)*	Nutritional/protective role (?)	Adult female (midgut)	Muturi et al., 2016
	*Culex pipiens* (wild samples)	**Nutritional role: larval development;** protective role (?)	Larvae	Steyn et al., 2016
Sand fly (Diptera: Psychodidae)	*Phlebotomus perniciosus*	**Protective role: killer strain with antifungal effect;** nutritional role (?)	Larvae (gut), adult male and female	Martin et al., 2016; Giovati et al., 2018
Honeybee (Hymenoptera)	*Apis mellifera*	**Protective role: immunomodulatory effect;** nutritional role (?)	Adult female (midgut)	Tauber et al., 2019
Plant hopper (Hemiptera)	*Laodelphax striatellus*	**Nutritional role: larval development;** protective role (?)	Nymphs, adult female (fat body)	Cao et al., 2015
Beetle (Coleoptera)	*Doubledaya bucculenta*	**Nutritional role: larval development;** protective role (?)	Eggs, larvae, and adult female (putative mygangium)	Toki et al., 2012, 2013

### Transmission Routes and Influence on Larval Development

Investigations in *An. stephensi* revealed the presence of *W. anomalus* in all the developmental stages of the mosquito (larvae, pupae, and adults). *Wickerhamomyces anomalus* is supposed to be associated with pre-adult stages also in *Ae. japonicus* and *Ae. triseriatus*, since it has been isolated from the midgut of adult females hatched in the laboratory from wild-caught pupae. Different results have been reported in *Cx. pipens*, which seems to host *W. anomalus* only at the larval stages, in fact, adult mosquitoes caught near the larval breeding sites tested negative for the yeast detection. As observed for most bacteria, structure and abundance of fungal communities vary during the mosquito life-cycle with a significant reduction of fungal diversity in the midgut of newly emerged adults (Steyn et al., 2016) as well as in females after blood ingestion (Muturi et al., 2016). Conversely, the presence of *W. anomalus* in newly emerged adults reared under controlled conditions supports the hypothesis of a trans-stadial transmission in *An. stephensi*, *Ae. japonicus*, and *Ae. triseriatus*. Except for the observations reported in *Cx. pipiens*, *W. anomalus* bypasses the fungal reduction during the mosquito metamorphosis from larva to adult, in different species. This is a prerogative of few microbes, which implies robustness and competitiveness in different habitats, since the pupal gut meet physiological adaptations that are needed for molting as hypoxia (Valzania et al., 2018).

*Wickerhamomyces anomalus* has been detected both in the midgut and gonads (male and female) of adults *An. stephensi*, suggesting its possible involvement in the mosquito reproduction and vertical transmission routes. Indeed, experiments performed using a monoclonal antibody targeting KTs demonstrated that *W. anomalus* is not affected by the blood meal and it is transmitted to the offspring (Cappelli et al., 2014). *Wickerhamomyces anomalus* maternal transmission through the egg surface in newly hatched larvae has been shown in the non-social beetle *D. bucculenta*, whose eggs acquire the yeast from the ovipositor-associated fungal pocket (mycangium) of adult females (Toki et al., 2013). This mechanism ensures an immediate acquisition of *W. anomalus* that is an essential food source for the larval development of *D. bucculenta*. A similar mutualism is proposed also in the planthopper *L. striatellatus*, in which *W. anomalus* favors the larval development and localizes the fat body of adult females, like other symbionts that are transmitted to the progeny through the ovary (Cao et al., 2015). Concerning mosquitoes, a positive influence of *W. anomalus* on larval survival and pupation has been shown in *Cx. pipiens*, using the yeast as an exclusive food source (Steyn et al., 2016).

### Role in Digestive Processes of Adult Insects

In adult mosquitoes, *W. anomalus* is supposed to establish trophic interactions useful to integrate a sugar restricted diet. This support has been described in *L. striatellatus*, in which *W. anomalus* provides nutrients to compensate the adult planthopper diet for the unbalanced composition of amino acids in plant phloem (Cao et al., 2015). Additionally, as floral nectar-residing yeast *W. anomalus* may support nutritional functions by acting on sugar digestibility. For instance, it may participate by fermentation to the breakdown of sugar while still residing on the flowers, as well as in the insect gut after the nectar collection, as suggested in *A. mellifera* (Tauber et al., 2019).

Another contribution of *W. anomalus* in digestive processes may be specifically exerted in adult female mosquitoes after the blood ingestion, since the yeast is among the few microbes that persist in the midgut after blood ingestion (Cappelli et al., 2019). This peculiarity is likely due to the ability of *W. anomalus* to adapt to different growth conditions, as a wide range of pH values and osmolarity. Indeed, chemical setup in the female midgut sensibly varies depending on the meal (sugar or blood), and during the blood digestion it changes from acid to basic pH values (del Pilar Corena et al., 2005). Moreover, *W. anomalus* withstands high concentrations of uric acid likely implying complete degradation pathways, thus it has potential to participate in the urate degradation and in the removal of nitrogenous wastes deriving from the blood digestion. Martin and collaborators demonstrated this contribution in females of the haematophagous diptera *P. perniciosus* by another insect-associated yeast, *Meyerozyma guilliermondii* (Martin et al., 2018).

### Antimicrobial Properties and Defense of the Host

In addition to nutritional support for larvae and adults, *W. anomalus* has been suggested for protective functions thanks to antimicrobial properties. It is demonstrated that yeasts can selectively shape the insect microbial community inhibiting the development of entomopathogenic fungi and increasing the development of mutualistic fungi (Davis et al., 2011). For example, *W. anomalus* defends larva-inhabiting bamboo internodes of *D. bucculenta* from the invasion of microbial contaminants, so that the inner surface of larval sites is covered with a fungal layer that represents a *W. anomalus* monoculture (Toki et al., 2012). However, the molecular basis of the antimicrobial activity in *D. bucculenta* has not yet been clarified. Instead, protective functions based on killer activities have been proposed in mosquitoes and the related mechanisms of action have been characterized (Cappelli et al., 2014). A strain of *W. anomalus* (F17.12) isolated from *An. stephensi* produces a KT with a strong antimicrobial activity likely exerting defense from entomopathogens. F17.12 secretes KT in specific cultural conditions as well as *in vivo* in the mosquito body, both in midguts and gonads (female and male). The KT-antimicrobial activity has been demonstrated *in vitro* against different susceptible yeast strains (e.g., of *Candida albicans*) and at diverse acidity conditions. Indeed, F17.12 KT activity assays showed that the yeast killer property spans a wide range of pH values (4.5–8). This finding fits well possible antimicrobial competition of *W. anomalus* in the mosquito body, since different physiological conditions occur in different organs (midgut and gonads). Likely in mosquitoes, a killer strain of *W. anomalus* displaying a killer phenotype and candidacidal activity has been isolated also in larvae and adults (male and female) of the sand fly *P. pernicious* (Martin et al., 2016; Giovati et al., 2018).

## Influence of *Wickerhamomyces anomalus* on the Mosquito’ Vector Competence

### Antiplasmodial Effects

On the basis that *W. anomalus* releases active KT in the female mosquito’s midgut even after the blood meal, the possible killer effect against the parasite in the mosquito midgut (early sporogonic stages) has been investigated. A strong antiplasmodial effect of KT has been demonstrated *in vitro* against early sporogonic stages (ookinetes) of the malaria rodent parasite *P. berghei*, with an inhibition of the parasite survival by 90%, and several morphological/structural alterations revealed by microscopy analysis (Valzano et al., 2016). These findings prompted additional *in vivo* studies highlighting that *W. anomalus* inhibits the development of *P. berghei* in the midgut of the malaria vector *An. stephensi* (Cappelli et al., 2019). Dietary supplementation of adult females with F17.12 reduced ookinetes in the midgut by 65%. *In vitro* and *in vivo* inhibition rates cannot be compared because of substantial differences between the two experimental systems. However, the result obtained *in vivo* is very important considering that the number of ookinetes that complete their development in malaria experimental models (e.g., *An. stephensi*/*P. berghei*) is considerably higher than those observed in wild mosquitoes (100-folder plus; Sinden, 1997). Therefore, *W. anomalus* has potential to significantly reduce the vector capability of mosquitoes in the field. Likely in mosquitoes, the yeast may also affect *Leishmania* (protozoan parasite) and phleboviruses in *P. perniciosus*, though further studies are necessary to evaluate anti-pathogen effects in the sand fly.

### Direct and Indirect Mechanisms of Action

Valzano et al. (2016) showed that effects on *P. berghei* ookinetes are due to direct implication of the KT-enzymatic activity. In particular, treatment with castanospermine, which is an inhibitor of exo-β-1,3-glucanase-mediated activities, reduced the killer effect on the parasite and the membrane damage. These data suggest that *P. berghei* death is induced by the hydrolysis of β-glucans located in the cell-wall of the parasite. The biochemical characterization of the KT purified from F17.12 identified a glycoprotein of 140 kD and limited electrophoretic mobility, corresponding to a high molecular weight β-glucosidase, as confirmed by activity tests in presence of castanospermine and the analysis of the predictive three-dimensional structure (Cecarini et al., 2019). Besides direct killer effects, yeasts can act by indirect mechanisms modulating the host immune system. For instance, *W. anomalus* has been reported as an active component of the gut with immunomodulatory effects in *A. mellifera* (Tauber et al., 2019). As regard mosquitoes, interesting results have been obtained by a dietary supplementation of *An. stephensi* with a KT non-producer strain of *W. anomalus* (UM3; Cappelli et al., 2019). The study demonstrated a reduction of the intensity of *P. berghei* infection by 30% (around half that by F17.12). Since UM3 is not able to induce any effect *in vitro* on cultivated parasites, indirect effects on the mosquito immune response are supposed. By the combination of direct KT-mediated activity and immune modulation of the host system, *W. anomalus* may sensibly affect the mosquito vector competence.

## *Wickerhamomyces anomalus* is a Promising Tool for “Symbiotic Control” of MBDs

Among a wide number of mosquito-associated yeasts (Malassigné et al., 2020), *W. anomalus* is a promising candidate for SC strategies against malaria and possibly other MBDs. We summarize main features that support this finding: (i) it is a mutualistic symbiont in *Anopheles*, *Aedes*, and *Culex* mosquitoes (impling a stable association with vectors); (ii) it bypasses the fungal reduction during the mosquito metamorphosis and after the blood ingestion; (iii) it spreads by trans-stadial and vertical transmission routes (impling self-spreading among mosquitoes and reducing efforts and costs of delivery); (iv) it inhibits the malaria parasite in the vector mosquitoes suggesting that, thanks to the wide killer activity, it has potential to affect a broad spectrum of mosquito-borne pathogens (e.g., arboviruses); (v) it is considered safe for humans and the environment (EFSA authorizes use of killer strains); (vi) it is clinically not relevant and its killer effect is based on exo-β-1,3-glucanase enzymatic activities (which targets microbes but is harmless in mammalian cell); (vii) it can be ingested by larvae and adults as a food source (advantage of yeasts over intracellular symbionts such as *Wolbachia*); and (viii) it can be formulated at low costs of production and released in the environment by dried-yeasts containing tablets, as recently carried out with fungal-larvicides used against *Aedes* spp. (Stewart et al., 2020).

## Concluding Remarks

Evidence of the ability of *W. anomalus* to inhibit the malaria parasite multiplication and survival in the mosquito, pose the bases for an exploitation of this killer yeast as an environmental-friendly and safe tool to interfere with vector competence. Since *W. anomalus* has been isolated in different vector species, further investigations are worthy for understanding whether it is able to interfere with different mosquito-borne pathogens. Its biological characteristics and the possibility of a large scale manifacture of a practical product both for storage and release, make *W. anomalus* a new yeast-based tool for “symbiotic control” strategies of MBDs.

## Author Contributions

AC and IR: conceptualization, original draft preparation, and editing. GF: manuscript reviewing. IR: project administration and supervision. All authors contributed to the article and approved the submitted version.

### Conflict of Interest

The authors declare that the research was conducted in the absence of any commercial or financial relationships that could be construed as a potential conflict of interest.
